# Reaching beyond the review of research evidence: a qualitative study of decision making during the development of clinical practice guidelines for disease prevention in healthcare

**DOI:** 10.1186/s12913-017-2277-1

**Published:** 2017-05-11

**Authors:** Linda Richter Sundberg, Rickard Garvare, Monica Elisabeth Nyström

**Affiliations:** 1 Department of Public Health and Clinical Medicine, Epidemiology and Global Health, Umeå University, SE 901 87 Umeå, Sweden; 2 Department of Clinical Science, Child and Adolescent Psychiatry, Umeå University, SE 901 87 Umeå, Sweden; 3 Department of Business Administration, Technology and Social Sciences, Luleå University of Technology, SE 971 87 Luleå, Sweden; 4 Department of Learning, Informatics, Management and Ethics, Medical Management Centre, Karolinska Institutet, SE 171 77 Stockholm, Sweden

**Keywords:** Clinical practice guidelines, Guideline development, Evidence-based policy-making, Group decision making, Prevention

## Abstract

**Background:**

The judgment and decision making process during guideline development is central for producing high-quality clinical practice guidelines, but the topic is relatively underexplored in the guideline research literature. We have studied the development process of national guidelines with a disease-prevention scope produced by the National board of Health and Welfare (NBHW) in Sweden. The NBHW formal guideline development model states that guideline recommendations should be based on five decision-criteria: research evidence; curative/preventive effect size, severity of the condition; cost-effectiveness; and ethical considerations. A group of health profession representatives (i.e. a prioritization group) was assigned the task of ranking condition-intervention pairs for guideline recommendations, taking into consideration the multiple decision criteria. The aim of this study was to investigate the decision making process during the two-year development of national guidelines for methods of preventing disease.

**Methods:**

A qualitative inductive longitudinal case study approach was used to investigate the decision making process. Questionnaires, non-participant observations of nine two-day group meetings, and documents provided data for the analysis. Conventional and summative qualitative content analysis was used to analyse data.

**Results:**

The guideline development model was modified ad-hoc as the group encountered three main types of dilemmas: high quality evidence vs. low adoptability of recommendation; insufficient evidence vs. high urgency to act; and incoherence in assessment and prioritization within and between four different lifestyle areas. The formal guideline development model guided the decision-criteria used, but three new or revised criteria were added by the group: ‘clinical knowledge and experience’, ‘potential guideline consequences’ and ‘needs of vulnerable groups’. The frequency of the use of various criteria in discussions varied over time. Gender, professional status, and interpersonal skills were perceived to affect individuals’ relative influence on group discussions.

**Conclusions:**

The study shows that guideline development groups make compromises between rigour and pragmatism. The formal guideline development model incorporated multiple aspects, but offered few details on how the different criteria should be handled. The guideline development model devoted little attention to the role of the decision-model and group-related factors. Guideline development models could benefit from clarifying the role of the group-related factors and non-research evidence, such as clinical experience and ethical considerations, in decision-processes during guideline development.

## Background

During the last decades, clinical practice guidelines (hereafter called guidelines) have become increasingly used tools for health care systems that aim to provide efficient and safe health care. Although guidelines have a common goal of informing health decision making based on patient preferences and the judgment of health professionals, the impact of guidelines has varied [1–4]. Challenging areas for achieving Evidence-based practice (EBP) are disease prevention and health promotion [5, 6], partially due to the time it takes to provide evidence of positive health outcomes, compared to most medical interventions [7].

Significant efforts have been put into increasing the trustworthiness, quality, and implementability of clinical guidelines [8–10]. The complexity of evidence and guideline uptake in health systems has been framed in the literature [11–13], and the potential of addressing determinants of implementation early in the guideline development process has been raised [8, 10, 14, 15]. Judgment and decision making processes during guideline development are central for high quality guidelines [16], but the group decision making involved has been relatively underexplored in the guideline research literature [17]. This study focused on a guideline development process during the phase of prioritizing and deciding on the best recommendations to be provided by national guidelines with a disease-prevention scope.

### Development of clinical practice guidelines

Development of high-quality guidelines involves technical, cognitive, and interpersonal processes [17]. Considerable efforts have been invested to strengthen technical aspects of guideline development (e.g. systematic collection and assessment of evidence) [18–21]. However, procedures for increasing guideline quality and making accurate use of evidence depend on human information processing, judgement, and sense-making, both individually and in groups. The core understanding of identical evidence can vary between groups [22], but this topic, compared to the technical aspects, has been sparsely explored empirically [23].

Guideline development generally includes identifying and refining the subject area, assembling panels, identifying and assessing evidence, translating evidence into clinical practice guidelines, and finally reviewing and updating the guidelines [16, 24]. Guideline development groups (GDGs) have often involved scientists and health professionals tasked with following explicit guideline development procedures [25, 26]. During the last decades, the emphasis on cost-effectiveness, ethics, and equity aspects to inform judgments and formulations of guideline recommendations has evolved [27–30].

Decisions about guideline recommendations have usually been made by a group of health profession representatives so as to assemble a wide range of knowledge, experiences, and opinions. Considerations made when recruiting individuals to such groups have involved variation in geographic representation, disciplines, stakeholder interests, gender, and different schools of thought [25, 31, 32].

Guideline development processes have typically been informed by manuals or models that share common features [8]. Recurring activities are to assemble a GDG, involve patients, identify clinical questions, perform systematic searches for and appraisals of research evidence, draft and finalize recommendations, and implement and continually update the guideline process [8]. Several studies have shown discrepancies between manual contents and the actual practice of guideline development [33–35] and manuals have been criticized for not being specific enough [36].

### Guideline development groups and decision making

The functioning of GDGs has been shown to influence how information is shared and processed and ultimately affects the guideline quality [37, 38]. In a study of 15 clinical guidelines on diabetes recommendations were found to differ significantly, even though they were based on similar evidence [39]. To avoid unwanted variation, guideline producers have aimed to instruct and direct the GDGs. However, how this steering has been operationalized in decision making models (e.g. through criteria, cues or vignettes) has varied [25].

For many patient populations and conditions evidence is limited [40]. To aid decisions on resource allocation, guidelines also need to involve value-based judgements. Consensus decision methods have often been used to define levels of agreement on controversial subjects [41], in particular the Delphi method, the nominal group technique and the consensus development conference [25].

The traditional Delphi method is characterized by iterations of individual decisions and group feedback to reach consensus, although participants do not meet with each other face-to-face and therefore can present and react to ideas unbiased by other participants [42, 43]. The Nominal Group Technique starts with individual generation of ideas that are discussed in a face-to-face group meeting and then statistically aggregated to form group judgements [44–46]. The consensus development conference involves one open and one closed chaired session. In the open session, evidence on a topic is presented by experts to a decision making group. In the closed session, the decision making group considers the issue in light of the data presented and aims to reach consensus [25].

Members of guideline groups tend to be biased in favour of procedures or theories linked to their field of knowledge or experience [47–49]. Higher levels of agreement more likely occurred if evidence was presented as a literature review or if the GDG consisted of a single profession (e.g. GPs) and the evidence was in accordance with group members’ beliefs [49]. Further, members’ willingness to reach consensus and their status in the group can outweigh evidence or other formal decision criteria when formulating guideline recommendations [50]. The chair of the group has the potential to influence processes and norms in a direction where information is distributed and shared [50, 51].

Group size and structure have been found to be relevant for the validity and reliability of guideline recommendations [52]. Between 6 and 15 multidisciplinary members has been suggested as the ideal group size for guideline development. Too few members might provide insufficient knowledge while too many might hinder effective group functioning [24, 50].

### The Swedish guideline development model

In Sweden, the National Board for Health and Welfare (NBHW) is responsible for the development of national guidelines. Guideline development is led by an executive board based at NBHW. The formal purpose of guidelines is to support and guide resource allocation within health and medical care and the social services system. Primary target audience is decision-makers within health care, while professionals, patients, and the public are secondary target groups.

NBHW guideline policies and instructions for guideline development state that rigorous assessment and use of scientific evidence should be the basis for guideline recommendations. Evidence is ranked by using the GRADE system (i.e. a formal method to categorise the quality of evidence and strength of recommendations in clinical practice guidelines) [18]. Guideline development is also affected by the Swedish Health Care Act that states that human dignity, patients’ needs, solidarity, and cost efficiency should inform all decisions involving resource prioritizations in health care, referred to as the ethical platform [53].

**Fig. 1:**
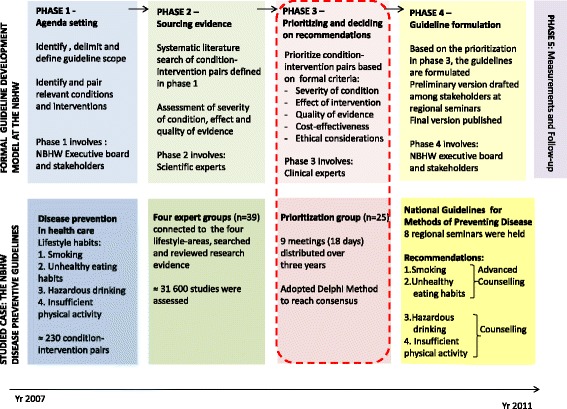
Development of national guidelines for methods of preventing disease – Formal model and studied case

Guideline development at NBHW consists of four phases and shares the characteristics of several other guideline frameworks [8]. The development process of the disease-prevention guidelines is illustrated in Fig. 1 and has been described in more detail in a previous study [15]. The first phase aims to define the guideline area (e.g. disease prevention). Within the guideline area, relevant health conditions (e.g. lifestyle habits such as smoking) are connected to remedial measures (e.g. motivational interviewing) in condition-intervention pairs. In the second phase, scientific experts perform a systematic literature search for studies on each condition-intervention pair. Evidence is assessed and graded using GRADE evidence assessment tool [18]. Further, health economists calculate cost effectiveness of the condition-intervention pairs. In the third phase, a group consisting of clinical experts (hereafter referred to as the Prioritization group, and group participants referred to as members) is assembled. The members rank the condition-intervention pairs based on quality of research evidence supporting the effect, the curative/preventive effect size, severity of the condition and cost-effectiveness.. The members also consider ethical implications according to the ethical platform. Ranking is done in two steps. In the first step, assessments are made individually and handed in anonymously. In the second step, all individual assessments are presented and discussed in the Prioritization group setting. The condition-intervention pairs are ranked on a scale from 1 (strongly recommended) to 10 (not recommended). The Prioritization group meetings are led by a chair and 1–3 representatives from NBHW national guideline department. During the fourth phase, the director general of NBHW decides on guideline recommendations based on the Prioritization group’s input. A preliminary guideline is distributed to stakeholders and target groups (e.g. decision-makers in the county councils) that provide feedback on applicability and potential organizational consequences. Based on analysis of this feedback, the final guidelines, including indicators for measurement of guideline adherence and patient-related outcomes, are produced. In the final fifth phase, the outcomes of guidelines are monitored and after 3–5 years the guidelines are up-dated.

Up until todayNBHW has developed 15 guidelines using the process outlined above.

Our focus is on the judgement and decision making process of the Prioritization group in the third phase of the NBHW guideline development model, i.e. prioritizing and deciding on guideline recommendations. Accordingly, the aim of this study was to investigate the bases for decisions and the decision making process of the Prioritization group during development of clinical guidelines with a disease preventive scope in Sweden.

Three more specific research questions were posed:

I. Which decision making criteria were used, and how did research evidence influence the Prioritization group’s judgment and decision making process?
II. Did the composition of decision criteria change over time, and if so, how?
III. Did the Prioritization group encounter conflicts or dilemmas during judgement and decision making? If so, on what subjects and how were these conflicts or dilemmas managed?

## Methods

### Key participants of the studied guideline development process

A qualitative, longitudinal, case-study approach was used to investigate decision making. In phase three (Fig. 1), the Prioritization group had to rank approximately 230 condition-intervention pairs related to area of disease prevention divided into four lifestyle areas: tobacco use, hazardous use of alcohol, insufficient physical activity and unhealthy eating habits. The Prioritization group members were selected by the NBHW based on their clinical expertise in relation to the preventive guideline scope. The number of persons that were contacted by the two representatives from the NBHW guideline executive board and the Chair via phone and email cannot be determined and the number of persons who refused or were unable to attend is not known.The experts included in the process explicitly declared no competing interests. The Prioritization group consisted of 25 members (16 women and 9 men) who represented different geographical areas, disciplines (e.g. psychiatry, obstetrics) and professional groups (e.g. physiotherapists, nurses). The Prioritization group participated in meetings to make judgments and reach consensus decisions for the selected 230 condition-intervention pairs (e.g. motivational interviewing for at-risk alcohol use). The meetings had a pre-set agenda that guided which clusters of condition-intervention pairs that were to be covered at each meeting. To finalize all condition-intervention pairs, the Prioritization group met during 18 days (at nine occasions, distributed over three years). Each meeting was led by a chair and two representatives from the NBHW guideline executive board (hereafter referred to as executive board members). Before each meeting the Prioritization group members individually rated each condition-intervention pair. Ratings were based on the criteria advised by the NBHW model for prioritization (i.e. condition seriousness, curative/preventive effect size, evidence supporting the effect, cost-effectiveness and ethical implications). The meeting procedure included presenting individual covert ratings for each such pair, followed by group discussions and open voting. This procedure was by the NBHW described as a modified Delphi-method since face-to-face group interactions were the key aspect of the studied process, an approach that differs from the traditional Delphi-methodology [25].

### Data collection

To answer the first research question an inductive approach was undertaken and data were collected from questionnaires, non-participant observations of meetings, and documents. For the second research question non-participant observations were the main data source. The third research question used data from observations and questionnaires. Two researchers (LRS and MN) conducted non-participant observations of 18 days of group meetings (Ʃ104 hours) using a semi-structured protocol indicating time and pre-set meeting agenda. Observational data contained aspects of verbal content (e.g. members’ verbal argumentation or the NBHW written agenda for the meeting) and the process (e.g. types of activities and procedures) and type of actor that made input (member or executive board members). Three questionnaires (Q1, Q2, Q3) were developed guided by the research questions and used to collect data on members’ experiences of the process. The questionnaires were distributed in connection to meetings, Q1 in the beginning (3^nd^ meeting, Sept 2009), Q2 in the middle (7^th^ meeting, March 2010), and Q3 at the end of the development process (9^th^ meeting, May 2011).

The questionnaires were anonymous and all three (Q1–Q3) contained a section with close-ended questions concerning socio-demographics (i.e. gender, education, occupation and employer). The reason for repeating these questions was to make analysis of the roles of these factors in each phase of the guideline development possible.In addition the questionnaires contained open-ended questions concerning experiences from the decision making process. Questionnaire number 1 (Q1) focused on members’ anticipation and experience of the initial engagement in the guideline development process (example of question in Q1: How did you prepare for participating in the prioritization group?). Questionnaire number 2 (Q2) focused on Prioritization group members’ experience of the NBHW model for prioritization and what decision criteria they based their decisions on (example of question in Q3: “Describe the principles for taking decisions in the prioritization group?”). Questionnaire number 3 (Q3) addressed Prioritization group members’ perception and experience of the decision making process in retrospect (example of question in Q3:“To what degree have you been able to communicate your opinions and knowledge in the group?”).

The questionnaires were distributed at the end of meetings. Response rates varied between 76% and 88% (*n* = 22, 19, 21). Missing data were due to participants not being present. 18 documents containing data concerning NBHW guideline policies (*n* = 4), instructions for guideline development (*n* = 11), the specific task description for the Prioritization group (*n* = 3) and the final versions of the NBHW National Guidelines for methods of preventing disease were collected. These documents provided background information and a description of the formal procedure and decision criteria to be used by the Prioritization group. In Table 1 an overview of the data collection is presented.

**Table 1:** Overview of data collection in relation to phases of the guideline development process (1–4)

Phase 1–2	Phase 3											Phase 4
2007–09	2009							2010			2011	2011–
	Jan	Mar	May	Jun	Sep	Oct	Nov	Jan	Mar	Apr	May	
	P1	P2	M1	M2	M3	M4	M5	M6	M7	M8	M9	
			O1	O2	O3	O4	O5	O6	O7	O8	O9	
					Q1				Q2		Q3	

*P* = Preparatory meetings – introduction of task

*M* Meeting 1–9, *O* Observations 1–9, *Q* Questionnaire (1–3)

(missing months = no data-collection occurred)

### Data analysis

Data analysis focused on identifying decision criteria used, variation in decision criteria over time, and types of dilemmas encountered (guided by research questions I–III). With these restrictions we used two approaches to qualitative content analysis; conventional and summative [54]. Conventional content analysis allows categories to emerge from data inductively and is used to capture the unique characteristics of the studied case and to allow for new understandings [54]. The conventional approach was used to analyse data from documents, questionnaires and observations, responding to research question I and III. The summative content analysis allows for the quantification of words or specific content in data sources and aims to illuminate the usage of a word or a group of words by analysing the frequency and the latent content of data [54]. The summative approach was used to analyse data from observations and to answer research question II.

The conventional analysis was conducted in several steps. First, the text in observation notes (*n* = 482 pages), questionnaires (*n* = 62 responses) and documents (*n* = 89 pages) was read through several times to get a sense of the material as a whole. In a second step, the text was divided into coherent text units and further analysed using software NVivo9. The text units contained information on how (e.g. discussions, behaviours, and statements) and on what basis (e.g. criteria or arguments) group decisions were made. In a third step text units with coherent statements (i.e. sentences and sections of text with coherent content) were coded with a condensed label intended to capture the essence of the unit’s meaning. Codes with a common meaning were then grouped into categories. In the fourth step, categories with a common meaning were merged into themes. The basis for and labelling of categories and themes were discussed among and decided on by two researchers (LRS and MN). Seven decision criteria were identified during the analysis. To assess the usage and development of various decision criteria during the two year prioritization process, we used summative content analysis [54] of the observational data. Synonymous expressions for the seven main decision criteria found in the conventional content analysis were identified. As an example of this, synonyms for the key term Research evidence were found in the terms *scientific base*, *scientific investigations and scientific arguments.* Key terms and their corresponding synonyms were then sought in the text through computer-assisted searches. Number of occurrences from each key terms and alternative term were summarized. Finally the occurrences of the terms were organized chronologically, indicating if they were expressed in the beginning (meetings 1–3), the middle (meetings 4–6), or the end (meetings 7–9) of the process in Phase 3.

The conventional and summative analysis was conducted by one researcher (LRS) and interpretations continuously discussed with another researcher (MN). LRS and MN were both aware of the content of the formal guideline development model.

## Results

**Fig. 2:**
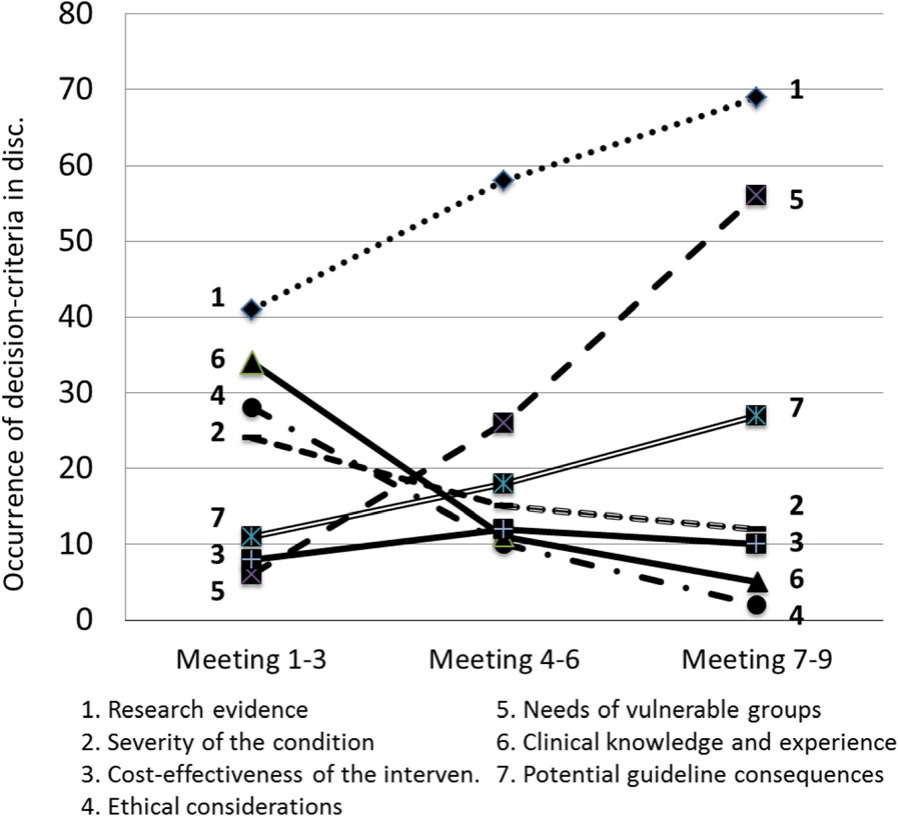
Decision making during guideline development – use of terms in the Prioritization group over time

The results are presented in three sections congruent with the three research questions: I) Decision criteria used by the Prioritization group II); the Decision making process over time; and III) Emerging dilemmas and related strategies. Results are summarized in Table 2 and Fig. 2.

**Table 2:** Themes and categories indicating decision criteria, dilemmas and outcome

Themes:	Formal and informal decision criteria	Additional factors influencing decisions	Emerging dilemmas	Decision outcome/consequence
Categories:	• Research evidence • Severity of the condition • Cost-effectiveness • Ethical considerations • Needs of vulnerable groups • Clinical knowledge and experience • Potential guideline and consequences	• Gender • Status • Verbal and Social skills	• High quality of evidence versus low adoptability of recommendations • Insufficient evidence versus high urgency to act • Incoherence in vertical and horizontal judgments	• For the Decision-process: -Formulate new principles for decision making -Re-appraise previous settled decisions or revise defined concepts -Additional search for evidence • For the Guidelines: -Intervention recommended in guidelines ranked 1–10 -Interventions recommended in a controlled clinical research setting -The intervention and/or condition is removed or integrated with another condition-intervention pair

### Decision criteria used by the Prioritization group

Group-decisions and judgments were found to be based on seven main criteria. The first four criteria are in accordance with the NBHW decision making model: 1) Research evidence, 2) Severity of the condition, 3) Cost-effectiveness of the intervention, and 4) Ethical considerations. The other three criteria emerged over time as an additional basis for decisions: 5) Needs of vulnerable groups, 6) Clinical knowledge and experience and 7) Potential consequences of guideline recommendations.There were also additional aspects that influenced the decision process: 8) Additional factors – Gender, social status, and interpersonal skills.

#### 1. Research evidence

To use of research evidence as a decision criteria in the prioritization process was a recurrent advice in the NBHW instructions for guideline development. The use of research evidence was also prominent in the observed group discussions and in the questionnaires (Q1, Q2, Q3). Observations indicated that when evidence was considered sufficient and clear the judgments were also highly consistent with individual ratings, resulting in a swift decision process where other criteria had little or no presence in the discussions. The Prioritization group members saw evidence as the superior criterion.

*“At first I tried to use all parts of the model. But now I have learned to use evidence as the main source in making decisions. I think the whole group has learned this.”* (Q3)

*“Ok, this is a given. The evidence is clear and the cost is relatively low. This is something we can recommend.”* (Observation meeting 8, Member)

The lack of evidence was a recurrent issue; discussed in the group meetings and also described in questionnaires (Q2, Q3). Even though over 31,000 research articles had been reviewed, several condition-intervention pairs were found to be insufficiently researched. Evidence was labelled as insufficient due to type of study (e.g. non-randomized single studies), too small effects or sample sizes, disparity between study population and guideline target population, disparity between study variables or outcomes and guideline operationalization of key concepts (e.g. risk for abuse of alcohol), and studies not being sufficiently detailed about the intervention or method for it to be translated into a guideline recommendation.

When evidence was lacking, the NBHW guideline development model recommended that the intervention should be tested and evaluated in a controlled clinical research setting or not be used at all. However, observational data showed that in some cases, when evidence was considered insufficient for conditions with highly prioritized target populations, other formal decision criteria, such as ethical considerations or the severity of the condition, became more prominent and/or new decision criteria (e.g. Needs of vulnerable groups) evolved in order to be able to recommend the intervention. The process to reach consensus then became considerably longer with postponed or repeated voting procedures and increased involvement of the chair and the NBHW guideline executive board. Arguments based on research evidence were associated with logic and rationality both by Prioritization group members and the executive board.

#### 2. Severity of the condition

Severity of the condition was a key component in the NBHW decision-model and served as a starting point for group discussions. This criterion refers to the potential risk of decreased life years or life-quality as a result of the condition.

Discussions about the severity of conditions were often linked to argumentation based other criteria, e.g. ethical considerations.

*“Even though evidence may be lacking, this is a very serious health condition. The ethical platform states that patients of greater needs should be given priority, so this is a difficult judgment.”* (Observation meeting 4, Member)

#### 3. Cost-effectiveness of the intervention

Observations showed that Cost-effectiveness of the intervention was – as the Severity of the condition-criterion – often used in combination with other criteria in the decision making process Cost-effectiveness was for example used as an argument to make criterion Research evidence or Severity of the condition, stronger or weaker.

*“A more time-consuming intervention is also more effective. But it is also more expensive. A short-term intervention is cost effective. It is also more ethical use of the patients’ time.”* (Observations, meeting 7, Member)

#### 4. Ethical considerations

Throughout the development process, we observed an ongoing debate about ethical implications of different guideline propositions. This theme was also elaborated on in questionnaires (Q3). Values and questions about right or wrong (in terms of behaviours, writings, and speech) were labelled by the Prioritization group as being ethical issues related to the role and obligations of NBHW, or to the group itself. Ethical considerations also concerned implications of guideline recommendations for health professionals or patients. Ethical reflections were divided into three main categories depending on their focus:

- Responsibilities as health professionals
  During the observed discussions the members raised ethical issues in relation to responsibilities to represent patients, based on their background as health professionals.
*“Even though we got this assignment from the NBHW authority, we must put the needs of the patients first.”* (Observation meeting 4, Member)
- Paternalism
  Discussions concerning the strive to acknowledge patients’ right to be informed about possible health risks of their lifestyle habits, but not wanting to be paternalistic and telling patients how to live their lifes.
*“If a person comes to the primary health care centre with depression or a sore throat, should he be asked questions about his eating habits? Is that the role of health care, to impose advice on people? Isn’t that a bit paternalistic?”* (Observation meeting 1, Member)
- Discussions referring the general ethical platform for prioritizations in health care
  Members presented arguments referring to patients’ equal rights to receive counselling to support changes in lifestyle.
*“If we as health professionals are well aware of the risks of unhealthy eating habits, do not our patients also have the right to know?”* (Observation meeting 4, Member)

Ethical discussions also addressed solidarity, i.e. that patient groups with greater needs and more severe suffering should be prioritized in the guideline recommendations. The need for prioritizing patients with greater needs became more obvious when interventions and conditions were compared. This discussion led the Prioritization group to construct an additional decision criterion – Needs of vulnerable groups.

#### 5. Needs of vulnerable groups

A perceived need to prioritize certain patient groups emerged during the observed group discussions and was described in questionnaires (Q2, Q3). The severity and consequences of a condition were assessed as higher and more far-reaching for certain groups and/or conditions (e.g. patients with cancer or hazardous alcohol consumption during pregnancy). Evidence was ranked as low for these groups when sample-sizes were small or when randomization had not been used.

*“When we divide the vulnerable groups in this way we will only find studies with low quality of evidence because the groups are so small. How can we recommend the best for these vulnerable groups?”* (Observation, meeting 4, Member)

The Prioritization group determined, via discussions with the NBHW management board, that certain defined vulnerable groups automatically would get a higher prioritization and stronger recommendation in the guidelines: patients preparing for surgery, pregnant or breastfeeding women, parents, and those with comorbidities. In the final guidelines, needs of vulnerable groups were defined as “increased severity of medical conditions due to certain circumstances” (NBHW, 2011, p. 19).

#### 6. Clinical knowledge and experience

One observed category of arguments was based on Prioritization group members’ previous experience and familiarity with specific interventions and/or health conditions. Some members used their clinical experience to stress the urgency of certain knowledge areas, perspectives, or patient groups. It was common to use clinical examples and express a patient perspective, especially during the first meetings. The Prioritization group members used hypothetical examples to test effects of the recommendations being discussed. However, the NBHW guideline executive board rejected the use of arguments based on clinical experience. These were considered to be subjective and expressions of opinion and, therefore, not valid as formal decision criteria.

*“This will be difficult to implement! I can imagine the challenge it would be to offer advanced counselling to all patients with unhealthy eating habits.”* (Observation meeting 2, Member)

*“But this is not the place for feelings.”* (Observation meeting 2, Executive board member)

*“Ok, I need to step back and consider the scientific base for this intervention.”* (Observation meeting 2, Member)

The executive board expressed contradictory signals to if and how the Prioritization group members should let their clinical experience influence judgments during guideline formulation.

*“Even though you are invited to this group (remark: the prioritization group) based on your clinical expertise, we want you to let your clinical experience to be subordinate to the guideline development model.”* (Observation meeting 2, Executive board member)

#### 7. Potential guideline consequences

A consideration that also influenced decisions was how guideline recommendations would be perceived and used by the target populations. This argument was observed in group discussions and also described in a questionnaire (Q3). The Prioritization group members tested different user perspectives, e.g. a physician at a health care centre or a local politician. The group anticipated discussions and reactions on the forthcoming guideline recommendations, especially from health care professionals. Endeavours to be consistent and trustworthy in the eyes of the target audience were reoccurring in the discussions.

*“I think we need to consider how this will be perceived among health professionals. What kind of signal do we want to send?”* (Observations, meeting 8, Member)

The above argument met some resistance among executive board members, who expressed that potential negative consequences of the guidelines should not limit the Prioritization group’s conclusions given that the guidelines to some degree had the role as a pioneer intervention to improve preventive health services.

#### 8. Additional factors – Gender, social status, and interpersonal skills

Additional factors that influenced discussions were also identified, mainly in questionnaire data (Q3) but also noted in observations. The Prioritization group members’ gender and status influenced group discussions. Male members and members of higher status in terms of education or age were perceived as having a greater influence on discussions. Verbal skills, timing, and ability to resist emotional expressions during discussions were also perceived by members as factors that led to greater influence. Abilities to be present, to listen, and to change one’s viewpoint were perceived as good strategies to achieve influence.

*“Being male, a good speaker, and having good rhetorical skills provided opportunity to influence. But listening and paying attention to other members’ arguments was also rewarding, if you wanted to influence discussions. Perhaps most crucial was to choose which questions you should invest in and save your arguments for the issues where you had knowledge.”* (Q3)

*“Influence was gained by grounding your arguments on facts and knowledge and staying away from becoming emotional.”* (Q3)

### The decision making process over time

The summative analysis indicated that the usage of different types of decision criteria in observed Prioritization group discussions varied in frequency over time. Figure 2 provides an overview of how the use of criteria evolved over time.

The NBHW executive board continuously interacted in the prioritization process by defining the meeting structure and providing directions to the Prioritization group. Arguments based on research evidence increased over time while arguments based on clinical experience declined by the middle of the process and expressed more seldom towards the end. This development was urged by the executive board members. The Prioritization group members expressed awareness about this variation and described it as constructive and as a learning experience.

*“It took me some time to really learn how to understand and use evidence in the discussions. In the beginning I was more emotional, thinking about how this intervention would work for me in my role as a physician.”* (Q2)

Terms related to 2) *Severity of the condition* and 3) *Cost-effectiveness* showed relatively stable patterns in occurrence over time. Arguments related to criterion 4) *Ethical considerations* were most frequent in the beginning and middle sections of the process and declined at the end. The added criterion 5) *Needs of vulnerable groups* was frequently used after it was established during the third meeting. Discussions on 7) *Potential guideline consequences* of the proposed recommendations increased in the later part of the process.

### Emerging dilemmas and related strategies

Several dilemmas emerged during the process, closely related to the variation of the discussion of different criteria over time. Three categories of dilemmas were identified, further described below.

#### Dilemma #1 – Evidence versus adoptability

The Prioritization group discussed potential implications of recommending interventions based on high quality evidence when these outweighed disease-preventive interventions with moderate (or low) quality of evidence already in use and widely regarded as successful. Some of the proposed interventions with high quality of evidence were extensive (i.e. demanding more time and skills) and they implied a wider gap in relation to current health care practises compared to some interventions supported by low-medium quality of evidence. Some Prioritization group members saw a risk of good-enough practices being abandoned in favour of recommended practices aiming for larger impact. As a solution, it was decided to be transparent about how the evidence was graded and used, and this was explicitly described in a specific section of the final guidelines.

*“This system has its downsides. When we recommend an intervention that we think is the best, we automatically undermine all other methods even though some of them show acceptable levels of evidence and that are working fine in the current preventive health practices. Health care will seek to implement the highest-ranked interventions, but if the gap is too wide and implementation fails, we are left with fewer preventive methods than before the guidelines.”* (Q2)

#### Dilemma #2 – Evidence versus urgency

The Prioritization group struggled with cases where the severity of the condition was assessed as very high, but where interventions supported by evidence were lacking.

*“This is a very serious health condition but the evidence is weaker. How do we merge these two aspects of the decisions?”* (Observation, meeting 2, Member)

Some of the Prioritization group members took the standpoint that *“it is better to offer something than nothing”* (Observation meeting no. 5). In these discussions an unwillingness to abandon methods that had insufficient evidence was expressed, and members argued for the need of recommendations even though evidence was insufficient, as there were no alternatives available. On the other hand, recommending methods that were supported only by low quality evidence was seen to potentially jeopardize the trustworthiness of the guideline process.

*“There is a risk that guidelines from the NBHW become an ideological megaphone that they are not taken seriously if we recommend methods without research evidence.”* (Observation, meeting 4, Member)

To avoid discriminating interventions targeting small vulnerable populations, it was decided to routinely increase the prioritization level one step of the recommended interventions for these groups. The purpose of this strategy was to make vulnerable groups visible and highly prioritized in the guideline recommendations. In some cases, vulnerable groups (e.g. pregnant women) were merged with other groups in order to aggregate the effects of the intervention. For new, not fully evaluated interventions where studies indicated promising results, the Prioritization group sometimes suggested further tests of the interventions in clinical research settings. A list of promising, but not yet evaluated interventions was formulated.

#### Dilemma #3 – Inconsistent judgments

The condition-intervention pairs were processed one at a time by applying the decision criteria. As the Prioritization group compared assessments of the interventions across lifestyle areas (tobacco use, hazardous use of alcohol, insufficient physical activity, and unhealthy eating habits) inconsistencies between some of the assessments were observed by the executive board and/or the Prioritization group members. To address this dilemma, the Prioritization group added an additional step in the decision process. When a decision was reached, the executive-board systematically compared it with decisions made earlier. If inconsistencies between prioritizations were found, the decision was reconsidered. On some occasions previous decisions were fully re-processed and new evidence (i.e. recently published scientific articles) was sought and found. When consistency was reached, a new principle for decision making was formulated by the Prioritization group and thereafter applied (i.e. *Needs of vulnerable groups*)*.*

## Discussion

This case study shows how the base for decision making during guideline development changes over time. This process-perspective on guideline development has been difficult to verify in the literature and therefore needs to be further investigated. Still it could be seen as a new approach to the well-studied phenomena of guideline development. The methodology we have used has some limitations (see the section for Methodological considerations) but we think that the process perspective has illuminated some important features of the guideline development process.

The NBHW guideline development model required that the condition-intervention pairs were to be prioritized based on research evidence; curative/preventive effect size, severity of the condition; cost-effectiveness; and ethical considerations. The Prioritization group used the formal guideline development model as a starting point for discussions. However, as the Prioritization group encountered dilemmas that the formal model did not cover (e.g. value conflicts) the decision model was modified accordingly, by adding new decision-criteria, new rules for decision making and/or by formulating additional writings in the final guidelines. This pragmatic use of research evidence will be discussed with a focus on group decision making, and the role of clinical experience and research evidence in guideline development.

### The formal decision model versus informal criteria and group influences

Information sharing processes may have been hindered as a consequence of several group-related circumstances (i.e. group size, group composition, consensus decision model) in the studied case. The studied Prioritization group (25 members) was larger than the ideal group size of 6–15 members [23], making it more difficult to involve all participants in discussions. The Prioritization group members’ gender, social status, and interpersonal skills were perceived to affect their relative contribution in group discussions. This finding corresponds well with several other studies [22, 45, 49, 50, 52]. The consensus decision model used was described as a “modified Delphi” by the NBHW but might also be viewed as an Estimate-Talk-Estimate process [55, 56]. This decision-making model made face-to face group-discussions highly influential for final decisions.

The observed deviation from the NBHW formal guideline development model is also in line with previous findings [33–35]. Most guideline manuals are generic outlines, intended for a wide range of guideline topics, target users, and populations [25, 31, 32]. The NBHW model is similar to other national guideline models in this sense [25, 32], and reflects a current trend of balancing rigour and pragmatism during guideline development [57]. Adhering to multiple criteria makes it harder for GDGs to reach consensus and the need for guidance by guideline development bodies, chairpersons, and explicit consensus-development methods could be expected to increase [24, 50], also exemplified in this study.

Accepted methodological standards for guideline development have been pushed to continuously higher levels of rigor [2, 4], with consequences in terms of costs, time, and usability of the guidelines [57]. As such, the tasks and demands for future guideline development groups will probably become even more complex, raising the question of the type of comptences and support guideline development groups will need.

### Research evidence versus clinical expertise and experience

Clinical expertise is emphasized in definitions of EBP, stating that EBP is about “integrating individual clinical expertise and the best external evidence” ([58] p. 312). In reality, EBP has been focused on generating, assessing, and making use of research evidence rather than on giving proportional attention to clinical experience and patient preferences [59]. Contemporary guideline development models (e.g. NBHW, NICE, WHO) underline the importance of stakeholder involvement in the process. One of the purposes of stakeholder involvement is to underpin guideline recommendations with the views and experiences of guideline target users (e.g. health professionals) [60].

In the studied case it was found that over time, research evidence out-triumphed arguments based on clinical knowledge and experience. The criteria *Research evidenc*e and *Clinical knowledge and experience* were contrasted in the Prioritization group’s discussions, but research evidence was almost always given a higher priority. The precedence of research evidence was underlined by the NBHW executive board and also supported by the NBHW instructions for guideline development.

The purpose of involving clinical experts in the guideline development process was rather vague and their expected contribution remained slightly unclear in the studied case. Diverse approaches to stakeholder involvement among guideline developers have also been seen in previous research. Most guideline developers involve stakeholders (e.g. patients or health professionals), in different stages of the process [61], mainly with the purpose of engaging future users of the guidelines and increase their quality with a broadened knowledge base [32]. Still, among leading guideline development agencies, such as NICE and WHO, clinical experts tend to play a minor role (e.g. comment on drafts, promote implementation) and they are not part of the voting or decisionmaking on the final guideline recommendations [62]. The ambivalence regarding the roles of clinical experts in previous research is also reflected within this case, where health professionals were involved based on their clinical expertise but where other actors tried to limit the influence of their clinical experience and knowledge in the decision process.

This tension between evidence and clinical experience and expertise is not often discussed in the guideline literature. How and to what extent clinical experience can be systematically identified and applied in guideline development remains unclear and is an essential area for further investigation.

### The prioritization process and its relation to implementation

Some of the key determinants of guideline adherence are settled already during guideline development (e.g. strength of recommendations, trialability, applicability, adoptability) [13, 15, 63]. Guideline manuals have also shown shortcomings in terms of information that could help prepare for guideline implementation, despite known organizational and system-level barriers [36]. Previous studies have investigated the possibilities to improve guideline implementability during guideline development [10, 12, 14].

The observed deviation from the pre-set NBHW decision-model is interesting from this implementation perspective as several of the emergent decision-criteria were connected to specific target users, target populations, postulated barriers, or guideline contexts that are linked to implementation determinant framework [63]. In the studied case, the Prioritization group added valuable perspectives to the NBHW decision model that potentially would aid implementation, such as accommodation (e.g. anticipated changes in workflow and professional roles), applicability (e.g. strategies to individualize recommendations based on clinical expertise), communicability (e.g. anticipated responses from health professionals and patients), and implementation (barriers and strategies).

The Prioritization group struggled to find a balance between recommending major changes to clinical practices based on best available research evidence and suggesting more feasible changes based on a combination of evidence, good practice, stated values of health care professionals, and existing disease-prevention health services. Adaption of a general pre-set decision model to fit the specific topic of the guideline and to the target users is inevitable and might also be beneficial. Adapting models to fit current settings and melding values, evidence, and costs into guideline recommendations might arguably increase the possibilities to successfully implement recommendations in health care organisations.

It is well known that clinical experience and research evidence sometimes are in conflict with each other [57, 59]. The results of our study are linked to recent debates on the role of methodological rigor in guideline development, where arguments for a balance between rigor and pragmatism, that invites stakeholders to negotiate criteria and adapt guidelines accordingly, have been put forward [57]. In this case our results show that when stakeholders are involved in guideline development in cases where the evidence is scarce a trade-off between validity and efficiency might occur. The Prioritization group struggled with a number of dilemmas related to the characteristics of research and implications for guidelines and target users. It took time and effort to solve the dilemmas, and a modified decision model was developed during the process. It could be questioned whether the implicit dilemmas in many of the discussions concerned differences in sources of input, i.e. research evidence, clinical experience, and patient preferences. Could this “rocky journey” have been foreseen, and could guideline models and manuals provide guidance on how to solve issues related to characteristics of evidence/research and adaptation to local contexts in the same spirit of transparency as for the methodological procedures? It could be argued that if the common dilemmas faced by guideline developers are not addressed, the use of guideline development models will not reach its full potential. In order to understand and improve guideline development processes further studies of decision making with a similar focus may provide guidance.

### Methodological considerations

This study examines only a part of the entire guideline development and implementation process – the process of prioritization when deciding on guideline recommendations. Thus, the reader should bear this in mind when thinking about the recommendations and suggestions made. Examining guideline development by observing it over time, documenting members’ experiences, and collecting documents did allow us to investigate the prolonged process of reasoning, judgment, and decision-making, and also permitted validation of findings from different sources. However, the methods used in this study have some limitations. Trustworthiness of studies is always important to consider. The observations are restricted by the ability of the observers to follow and document the process. Observations could have been improved by using video recordings, but the large number of members and the characteristics of the premises made this very difficult. In this study two researches were involved in observations (4 of 9 meetings) and analyses in order to reduce bias and shortcomings. Furthermore, it was presumed that group decisions were based on arguments explicitly articulated during meetings and/or indicated by members in questionnaires. The increase (or decrease) of an articulated argument over time might not entirely reflect its role in the final decision and should be interpreted with caution. It was though considered to be an acceptable indicator of how decision criteria were used over time.

## Conclusion

This study of group decision making during guideline development illustrates that guideline development is a complex and dynamic process.

The studied case has shown that GDGs make more or less transparent compromises among research evidence, clinical experience and ethical considerations. The emphasis on these criteria also varies over time in Prioritization group’s discussions. Guideline development models are often detailed concerning how research evidence should be identified and ranked. However, the model offered few details on how other criteria (e.g. severity of the condition, ethics) should be handled in group decisions, particularly when the research evidence was sparse or ambiguous. Aiming to solve the dilemma of integrating preferences, the practical field of guideline development could benefit from providing more details on how this could be made possible and transparent.

To obtain comprehensive advice on generic dilemmas that GDGs encounter, further research on the strategies used and the processes that take place within guideline development is needed, along with assessment of how guidelines that more or less take clinical expertise and experience into account are translated and used in different context.

## Abbreviations

EBP: Evidence-based practice
GDG: Guideline development groups
NICE: The National Institute for Health and Care Excellence
NBHW: The National Board of Health and Welfare
WHO: World Health Organization
